# Bicentennial of Gregor Johann Mendel’s birth: Mendel’s work still addresses geneticists in 2022

**DOI:** 10.3389/fpls.2022.969745

**Published:** 2022-09-07

**Authors:** Boris Vyskot, Jiri Siroky

**Affiliations:** Department of Plant Developmental Genetics, Institute of Biophysics of the Czech Academy of Sciences, Brno, Czechia

**Keywords:** Gregor Mendel, hybridization, heredity, plant science, history

## Introduction

Two hundred years ago, on 20 July 1822, Gregor Johann Mendel was born in the small Silesian town of Heinzendorf bei Odrau at that time a part of the Austrian Empire, now Hynèice in the Czech Republic. Members of the scientific world, as well as the general public, are looking upon the bicentennial as a chance to gain new insights into Mendel’s work. Surprisingly, interest and acclaim for Mendel were not immediately forthcoming despite him making the authorities acquainted with his experimental findings on plant hybridization in 1865–1866. It remains a closed book as to why Mendel’s carefully composed publication was unappreciated for more than 30 years. The only exception from the scientific community in the 1860s was Carl von Nägeli who exchanged unproductive correspondence with Mendel regarding his work on plant hybridization of the garden pea (*Pisum sativum*).

## Mendel’s background and his findings

From 1856 to 1863, Mendel experimentally crossed hundreds of plants, the majority of which were the garden pea (*Pisum sativum*). His findings were cautiously evaluated, and his *Pisum* work was presented in two lectures given on 8 February and 8 March 1865 at meetings of the Natural Science Society of Brno. Moreover, he summarized his findings in Society’s Proceedings in a carefully prepared and detailed article in 1866 and sent this publication to institutions and botanists across Europe. Nevertheless, his findings did not produce any notable response with the only exception being Carl von Nägeli, a Swiss-born Professor at the University of Munich. Unfortunately, according to Mendel’s letters (Mendel, 1950), Nägeli had “mistrustful caution” regarding his hypotheses deduced from *Pisum* work. Besides the study of “variable hybrids” (*Pisum*), Mendel also studied “constant hybrids” using various *Hieracium* species as model plants. This part of Mendel’s work was highly appreciated by Nägeli as can be seen from mutual correspondence. Mendel accomplished his *Hieracium* work in 1870, and the results were published (Mendel, 1870).

Now, let us examine the reasons why Mendel’s findings on the hybridization of the garden pea were almost universally ignored by his contemporaries: Did Mendel make a mistake when publishing his work in local Proceedings in 1866? Was there any chance for him to communicate his results in a respected scientific periodical? What was the situation with scientific journals in Mendel’s time? And what was the scholarly milieu in Brno in the mid-19th century?

The present study first looked at Mendel’s background. He was certainly well educated and undeniably gifted. From 1840 to 1843, he studied philosophy and physics at the Philosophical Institute of the University in Olomouc, and along with languages (Greek and Latin), he was attracted to natural sciences. Perhaps more due to financial rather than spiritual reasons, Mendel commenced in Augustinian St. Thomas’s Abbey in Brno in 1843 in order to continue his education and became a friar in 1847. As part of their monastic duties, he was usually expected to teach at local grammar schools or similar educational facilities, so he was sent to the University of Vienna to acquire additional skills in physics and mathematics. Mendel spent 2 years (from 1851 to 1853) in Vienna attending courses of famous professors such as the accomplished physicist Christian Doppler and the well-known mathematician Andreas von Ettingshausen. Mendel clearly benefitted from the training in the scientific methodology of experimental work, as he demonstrated later during the research conducted in Brno (Orel, 1984, 1996).

Despite the fact that Mendel presented his results on “Experiments with Plant Hybrids” giving two public lectures in Brno, published in a local natural periodical, and notably, promoted his work by sending his article to well-known biologists knowledgeable about natural history, his discoveries were mostly ignored. The dissemination of Mendel’s findings through lectures and/or publishing in “Proceedings of the Brno Society for the Study of Natural Science” (Mendel, 1866) during the 1860s was adequate and common; thus, we must discard the objection of it being a poor decision to choose the “Scientific Journal” (Schwarzbach et al., 2014).

Obviously, in the mid-19th century, there were many distinguished scientific journals, e.g., Philosophical Transactions of the Royal Society (issued from 1665) and Comptes rendus des séances de l’Académie des Inscriptions et Belles-Lettres (issued from 1857) to mention the oldest and most famous. Moreover, scientific periodicals such as we see in our time were *sensu stricto* not scientific or non-existing in Mendel’s era; even Nature was only published in 1869, 3 years after Mendel shared his findings in writing. Also, was Mendel aware of the importance of reporting his research in an internationally recognized journal as we call these periodicals today? The question here is if Mendel considered his work as a discovery or simply as a means of communication of his observations.

While there is a general belief that Mendel was not understood by his audience during lectures presented at the meetings of the Brno Natural Science Society and that there was practically no discussion from attendees; the opposite is true, as stated by Moore (2001), both talks were greatly appreciated according to the local newspapers at the time. Brno, however, was to some extent more of a cultural center and profited from the textile industry during the 19th century, rather than being an educational hub. In fact, there was no university-type institution in the city until 1849 when the Apprentice Technical Training Center was established, and eventually converted to Academe in 1873. So, unfortunately, Brno did not provide many similarly minded intellectuals to support nor even acknowledge Mendel’s ground-breaking work. According to Orel (1984), the only person from Mendel’s neighborhood who was interested was the Abbot of the Monastery, Cyril Napp who recognized the significance of his research and its potential application to the monastery’s extensive agricultural holdings in Moravia. It was also Napp who was responsible for Mendel being able to study in Vienna. It is perhaps rather disheartening that others were not so open to valuing Mendel’s diligent efforts.

Also of note, Mendel’s method of planning experiments and evaluating the results of hybridization was new, unusual, and surprising for that era; it was exactly what we call “hypothesis-driven research” today. While this innovative approach was quite exceptional in Mendel’s time, it is how contemporary experimental science operates nowadays. One common misunderstanding regarding his analyses however is: “Why did Mendel not use the established statistical approach of the chi-square test which is the first-choice method in evaluating progeny phenotype ratios?” The answer is simple; this statistical method (Helmert, 1876) was not formulated and published until 10 years after Mendel released his results.

Sadly, Mendel did not receive the recognition he deserved until more than three decades had passed. In the year 1900, three independent scientists, Correns, DeVries, and von Tschermak, experimenting with plant hybrids and studying the literature came across Mendel’s paper. Actually, Correns run across Mendel’s Versuche even in 1896 conducting his ample hybridization work (Rheinberger, 2015). These three botanists, independently, formulated Mendelian Laws of Inheritance, something that Mendel never did. In the interpretation of Mendel’s work, we often meet with some fables about what Mendel discovered. As an example, Mendel worked with symbols like *A, a*, and *Aa*. In his theories, these letters of the alphabet represented *character* (in German *Merkmal*), something one can clearly recognize in plant morphology, seed shape, etc. *A* denotes one character, *a* denotes a contrasting character, and *Aa* represents the hybrid nature of the progeny. As these are today attributed as symbols representing genes or alleles, the use of the word *characters* misleadingly implies that Mendel formulated Mendel’s Laws (at least one of them, the Law of Segregation). In the conclusion of Versuche (Mendel, 1866), summarizing his findings, he used abundantly the word *Elemente* which represented unknown substances that might produce *Merkmal.* By simplification, it can be deduced that Mendel was very close to the gene concept, but this cannot be inferred from his paper. Similar myths can be found elsewhere, even in genetics textbooks.

Certainly, the discovery of chromosomes and the formulation of the Chromosome Theory of Inheritance by Sutton (1903) and Boveri (1904), again independently, constituted the milieu for the enrichment of new exploding biological disciplines (see Maderspacher, 2008, for a comprehensive review). By comparing numerous texts emerging since the rediscovery of Mendel’s work with the hybridization of plant species in 1900, we see continuous reference to genetics, population genetics, molecular genetics, medical genetics, and even epigenetics.

At the end of Mendel’s scientific career with *Pisum* hybrids, he continued with the work on interspecies hybrids, crossing bees, conducting meteorological observations, and more. In 1868, Abbot Napp died and Mendel opted for a new Abbot. He then accepted many honorable positions, e.g., the position of Director of the Moravian Mortgage Bank, Chair of Naturforschenden Verein in Brünn, and member of the Association of Moravian Beekeepers.

## What Mendel could not know

Due to Mendel’s laborious selection, he achieved conclusive results in *Pisum* work, which were presented in his lectures and article (Mendel, 1866). When working with *Hieracium* constant hybrids (Mendel, 1870), he really obtained true hybrids using the very arduous method of operating on a single floret under a microscope almost losing his sight (Van Dijk and Ellis, 2016). However, *Hieracium* is an apomictic plant and seed progeny is identical to maternal genotype. This fact was not known in Mendel’s times.

Traits can be classified according to their expressivity and penetrance. Expressivity is the extent of manifestation of the trait, while penetration is the probability of the transfer of the trait to the next generation. The pea markers he selected had full manifestation as well as complete penetration and were encoded by single genes which were not in a closed linkage. Now, we know that the penetrance and expressivity of genes are mainly controlled by epigenetic processes. Epigenetics is a new branch of genetics that deals with the regulation of gene function. It means that genes are not “naive” but their expression is controlled by chromatin modifications. This is the basis of the adaptive processes which organisms can use to react to a changing environment. There are several chemical processes involved in the regulation of gene function. They mainly include DNA cytosine methylation, histone modifications, and RNA interference (for review see Ferguson-Smith et al., 2009). They work in accord, controlling gene function and finally the resulting phenotype. Although it is sometimes stated that these chromatin changes are not stable and are reversible, it is not always the case. We are now in the era of molecular biology and so can answer some of the questions that Mendel’s foundation laying work raised in the middle of the 19th century.

Interestingly, there are at least two (epigenetic) exceptions that do not follow Mendel’s laws. The first one stated that alleles segregate independently. This is not valid in many cases when epialleles (i.e., genetically identical but epigenetically distinct alleles) in heterozygotes mutually interact leading to a heritable change in their expression (paramutations). The second law says that reciprocal crosses are identical without any influence on the sex of parents. This is not valid in the case of parental imprinting in which some loci are in gametogenesis sex specifically modified which leads to their silencing in the filial generation.

**Figure:**
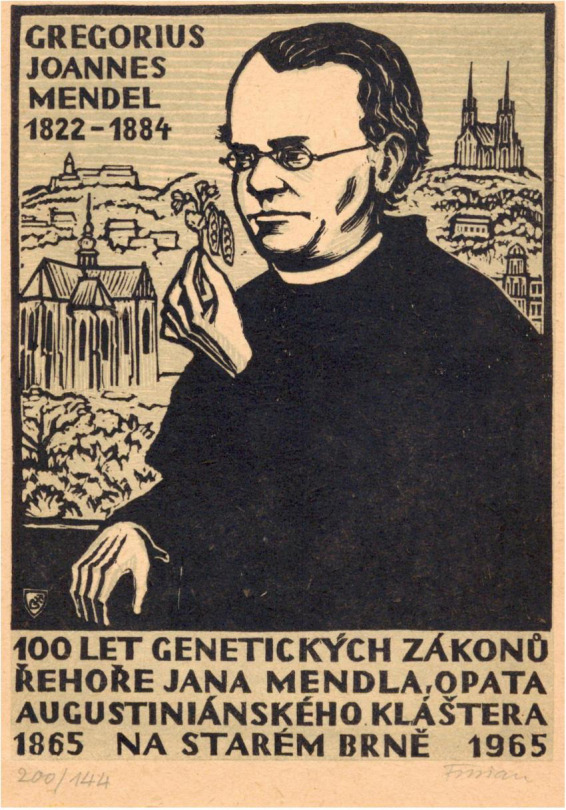
Gregor Johann Mendel and his work have been celebrated on various occasions. Woodcut by Michael Florian issued at the centennial of Genetics Laws. Text in Czech states: 100 years of Genetic Laws by Gregor Johann Mendel, Abbot of Augustinian Monastery in Old Town of Brno.

## Conclusion: Way ahead in science

Gregor Johann Mendel was ahead of his time and applied mathematics in biology. No one before him used such exact analyses in biology. It also could be a reason why his contemporary colleagues did not understand his research and thus it was undervalued. It was rediscovered at the beginning of the 20th century when Dutch botanist and geneticist Hugo DeVries, German geneticist Carl Correns and Austrian agronomist Erich von Tschermak (reviewed by Roberts, 1929) independently confirmed Mendel’s laws. Since that time Mendel has been re-evaluated as the founder of genetics. Even after 150 years, the basic principles of genetics as discovered through Mendel’s experiments are still vital and relevant to today’s research.

## Author contributions

Both authors contributed to manuscript revision, read, and approved the submitted version.
